# One-Pot Synthesis of Silicone–Urethane Hybrid Foam and Comparison of Flame Retardant, Rheological, and Mechanical Properties with Polyurethane Foam

**DOI:** 10.3390/polym17172352

**Published:** 2025-08-29

**Authors:** Sosan Hwang, Hyeon Woo Jeong, Asell Kim, Tae Soon Kwan, Sun Kyoung Jeoung, Sung-Hyeon Baeck, Sang Eun Shim, Yingjie Qian

**Affiliations:** 1 Guangzhou Institute of Energy Conversion, Chinese Academy of Sciences, NengYuan Street 2, Tianhe District, Guangzhou 510640, China; shwang@katech.re.kr (S.H.); 22201563@inha.edu (H.W.J.); 2 Department of Chemistry and Chemical Engineering, Education and Research Center for Smart Energy and Materials, Inha University, Incheon 22212, Republic of Korea; 3 Chassis & Materials Research Laboratory, Advanced Materials R&D Department, Korea Automotive Technology Institute, Cheonan 31214, Republic of Korea; 4 Korea Railroad Research Institute, Uiwang-si 16105, Republic of Korea

**Keywords:** polyurethane foam, silicone foam, hybrid foam, tin octoate, flame-retardant, viscoelasticity

## Abstract

This study presents the design and fabrication of silicone–urethane hybrid foam (SUF) to improve fire safety in transportation seating. Tin(II) 2-ethylhexanoate (Sn(OCT)_2_) was used to catalyze reactions between bifunctional isocyanates, polyols, and vinyl-terminated PDMS, enabling simultaneous curing and foaming. Sn(OCT)_2_ effectively facilitated both the foaming and gelation processes of silicone and urethane chemistries. The resulting SUF demonstrated a 44.55% reduction in peak heat release rate (PHRR) compared to UF, due to the PDMS network’s synergistic flame-retardant and barrier effects. Additionally, the crosslinked PDMS structure maintained strong mechanical integrity. This study offers a simple and effective approach for producing SUF with enhanced fire safety.

## 1. Introduction

Polyurethane foams (UFs) are widely utilized as thermal insulation and cushioning materials due to their low thermal conductivity, excellent mechanical properties, and low density [1,2]. Typically, UFs are produced by reacting polyol (a hydroxyl compound) with monomeric or polymeric 4,4′-diphenylmethane diisocyanate (MDI) [3] and formed through the reaction of MDI and H_2_O [4]. However, the flammable hydrocarbon components and permeable structures cause UFs to ignite rapidly, releasing heat and toxic gases, which limits their use [5]. Thus, development of UFs with intrinsic flame-retardant and smoke-suppression properties is highly desirable. Furthermore, the widespread application of UFs necessitates eco-friendly flame retardants to meet fire safety standards [6,7].

**Figure 1:**
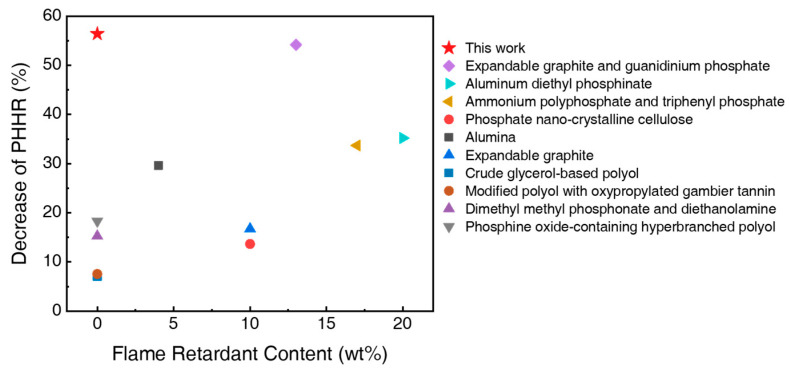
Comparison of the reduction in PHRR and filler content of SUF with previously reported results for fire-resistant polyurethane foam. (Expandable graphite and guanidinium phosphate [8], Aluminum diethyl phosphinate [16], Ammonium polyphosphate and triphenyl phosphate [17], Phosphate nano-crystalline cellulose [18], Alumina [19], Expandable graphite [20], Crude glycerol-based polyol [21], Modified polyol with oxypropylated gambier tannin [22], Dimethyl methyl phosphonate and diethanolamine [23], Phosphine oxide-containing hyperbranched polyol [24]).

Figure 1 compares the reduction in the peak heat release rate (PHRR) relative to flame retardant content. The flammability of UFs can be suppressed by adding flame retardants or using modified polyols [8]. Flame retardants with halogen [9,10], phosphorus [11,12,13], and nitrogen [14,15] are effective; however, the use of halogen-based retardants is discouraged. Consequently, flame-retardant UF is prepared using phosphorus-based flame retardants such as aluminum diethyl phosphinate [16], ammonium polyphosphate [17], triphenyl phosphate [17], and phosphate nano-crystalline cellulose [18]. Additionally, alumina [19] and expandable graphite have been employed to fabricate flame-retardant UFs [20]. Moreover, modified polyols have been incorporated into UFs to enhance performance [21,22,23,24]. For example, Hejna et al. utilized a crude glycerol-based polyol to reduce PHRR [21], Arbenz et al. modified polyol with oxypropylated gambier tannin for thermal stability [22], Rao et al. fabricated UFs with a novel polyester polyol synthesized from dimethyl methyl phosphonate and diethanolamine to decrease PHRR [23], and Ma et al. applied a novel phosphine oxide-containing hyperbranched polyol to UFs [24]. Although various studies have been conducted on flame retardants, common flame retardants still suffer from issues of toxic gas emissions and reduced mechanical properties [8].

Silicone polymers, known for their unique molecular structures and outstanding physical properties, have gained significant industrial and academic interest. Specifically, polydimethylsiloxane (PDMS) with its stable Si-O-Si backbone offers excellent mechanical flexibility, weatherability, broad temperature stability, and superior electrical resistance [25,26,27]. Carbon-based polymers blended with PDMS exhibit enhanced thermal properties and flexibility [28]. The hybrid material attains excellent mechanical strength and stability through entanglement, due to the high crosslinking density of PDMS. Also, the addition of hydroxyl-terminated PDMS during the fabrication of UFs improved their thermal and mechanical properties [29]. Dibutyltin dilaurate (DBTL) has been used as a catalyst in the preparation of UFs. However, due to its toxicity and environmental impact [30], the use of DBTL has been discouraged since the early 2000s [31]. The FDA has banned DBTL for use in flexible foams, leading to its replacement with tin (II) 2-ethyl hexanoate (Sn(OCT)_2_). It has been known that Sn(OCT)_2_ is involved in reactions between hydrogen-terminated PDMS (H-polymer) and hydroxide-terminated PDMS (OH-polymer) [32] in silicone chemistry and may also be applied to silicone–urethane hybrid composites.

In this study, we investigated the world’s first one-step synthesis of SUF and examined the catalytic role of Sn(OCT)_2_ in both the foaming and gelation processes. Although Sn(OCT)_2_ cannot directly participate in the crosslinking of vinyl-terminated PDMS (vinyl-polymer), we found that it effectively promotes the crosslinking of vinyl-polymers with isocyanates. This discovery enables the production of silicone–urethane hybrid foam (SUF) with exceptional durability and flame-retardant properties. For the first time, we propose a novel chemical reaction pathway for Sn(OCT)_2_ as a catalyst that facilitates the crosslinking of vinyl-polymer. The primary objectives of this research are to understand the catalytic mechanism of Sn(OCT)_2_ and to investigate the superior various properties of SUFs.

## 2. Experimental Section

### 2.1. Materials

Vinyl-polymer (RFVN-0100, 100,000 cps, Mw 140,000) and H-polymer (RFHD-3003, 17.3 cps, Mw 2000) were purchased from KBG (Cheonan City, Chungcheongnam-do, Republic of Korea). OH-polymer (DMS31, Gelest, Morrisville, PA, USA, 1000 cps, Mw 26,000) used in this study is a dual-terminal polymer. The catalyst employed for crosslinking and hydrogen gas generation was Sn(OCT)_2_ (SUT-9, SD-Korea, Hwaseong City, Republic of Korea, 1000 cps). For the synthesis, FlexFoam-iT!™ III from Smooth-On, Inc. (Macungie, PA, USA) was utilized for UF, and FlexFoam-iT!™ IIIV from Smooth-On, Inc. was used to compare their properties (denoted as UF*).

### 2.2. Preparation of SUF

To prepare SUF, vinyl-polymer, OH-polymer, Part B (including polyol) of urethane, and Sn(OCT)_2_ were mixed with a hand mixer for 2 min. Following this, H-polymer and Part A (including isocyanate) of urethane were added to the mixture and mixed for an additional 30 s The resulting mixture was quickly poured into a mold, and primary curing was performed in an oven at 100 °C for 30 min. Four types of SUF were prepared by varying the polyurethane content in silicone with their composition ratios enlisted in Table 1.

For UF, FlexFoam-iT!™ III (Part A:Part B = 57.5:100) was mixed for 1 min, poured into a mold, and foamed in an oven at 60 °C for 30 min. Additionally, FlexFoam-iT!™ IIIV (Part A:Part B = 100:100) was mixed for 1 min, poured into a mold, and foamed in an oven at 60 °C for 30 min, resulting in UF*. The SF for the repeated compression test was prepared by mixing vinyl-polymer, H-polymer, and OH-polymer in a ratio of 6:2:1, and then foamed in an oven at 80 °C for 30 min using Karstedt’s catalyst. Subsequently, secondary curing was performed in an oven at 100 °C for 12 h.

### 2.3. Characterization

The crystallinity (*X_c_*) of SUF was assessed using multipurpose X-ray diffraction (XRD, Pro MRD, Malvern Panalytical Ltd., Malvern, UK). SUFs were characterized by thermogravimetric analysis (TGA, TGA 4000, Perkin Elmer, Waltham, MA, USA) under a nitrogen atmosphere at heating rates of 10, 15, and 20 °C/min and heated to 900 °C to investigate their thermal properties. In addition, the residual amounts of -NCO and Si-H were confirmed by Fourier-transform infrared spectroscopy (FT-IR, Spectrum Two, Perkin Elmer, Waltham, MA, USA). Moreover, differential scanning calorimetry (DSC, DSC8000, Perkin Elmer, Waltham, MA, USA) was used to compare *T_m_* (melting temperature), *T_c_* (crystallization temperature), and enthalpy of SUF at a heating rate of 10 °C/min within the range of −125 to 0 °C. The degree of *X_c_* for each specimen was determined using the following equation (Equation (1)).

(1) $$X_{c}\left(\%\right)=\frac{{∆H}_{f}}{{∆H}_{f\;}^{0}w}$$

where $∆H_{f}$ represents the heat of fusion of SUF, ${∆H}_{f}^{0}$ denotes the heat of fusion of the neat PDMS with a *X_c_* of 100% (61.19 J/g), and *w* is the mass fraction of the neat PDMS in the SUF [33]. Additionally, 1H nuclear magnetic resonance (^1^H-NMR) spectra were utilized for the structural characterization of the SUFs. The NMR spectra were acquired using a 700 MHz NMR spectrometer (DD2 700, Agilent Technologies, Santa Clara, CA, USA) with deuterated chloroform as the solvent.

### 2.4. Mechanical Properties of SUF

The mechanical properties evaluated included tensile strength, elongation at break, and compressive strength. The tensile and compressive strength tests are conducted in accordance with ASTM D3574. The cross-sectional area of the specimen for the tensile tests was 25 × 25 mm^2^, and the gauge length was 40 mm. Tensile tests were conducted at a speed of 50 mm/min using a universal testing machine (UTM; DUT-1TCM, Daekyung Engineering, Republic of Korea) equipped with a 200 N load cell. The compression test was performed at a speed of 5 mm/min, with the specimen having a cross-sectional area of 50 × 50 mm^2^ and a load cell of 2000 N.

### 2.5. Thermal Properties of SUF

A cone calorimeter test was conducted on sheets with dimensions of 100 × 100 × 50 mm^3^ following the ISO 5660-1 standard [34] and a heat flux of 25 kW/m^2^. The data obtained from the cone calorimeter represents the average of triplicate measurements. 5 to 10 mg of SUFs were used to obtain TGA curves under a nitrogen atmosphere at heating rates of 10, 15, and 20 °C/min, with temperatures raised to 600 °C. The derivative differential thermal analysis-thermogravimetry curves were subsequently calculated. The activation energy of SUF was determined using the Horowitz-Metzger equation (Equation (2)). *W*_0_, *W_f_*, *W_t_*, *E_ac_*, *R*, *T_m_*, and *θ* represent the initial weight of the sample (g), final weight after thermal decomposition (g), weight at each temperature (g), activation energy (kJ/mol), gas constant (8.3145 J/mol·K), temperature at the highest pyrolysis rate (°C/min), and *θ* = *T_m_* − *T*, respectively [35].

(2) $$ln\left[ln\frac{w_{0}-w_{f}}{w_{t}-w_{f}}\right]=\frac{E_{ac}\theta }{RT_{m}^{2}}$$

### 2.6. Viscoelastic Properties of SUF

The rheological properties of SUFs and UF were investigated using rubber process analysis (RPA, RPA-V1, U-CAN Dynatex Inc., Taichung, Taiwan). This analysis employs a rotorless biconical die design consisting of two cone-shaped dies. During testing, the lower die oscillates the sample sinusoidally according to preprogrammed strains at a controlled temperature, while the upper die, connected to a reaction torque transducer, measures the complex torque (S*) and the phase angle transmitted through the sample by the lower die. Following the ASTM D6204 standard, the pure elastic torque (*S*′) and pure viscous torque (*S*″) were determined from the complex torque and phase angle. From these measurements, the elastic shear modulus (*G*′) and viscous shear modulus (*G*″) can be calculated based on the applied strain, as follows:

(3) $$G^{\prime }=KS^{\prime }/Strain$$

(4) $$G^{\prime \prime }=KS^{\prime \prime }/Strain$$

where *K* is a constant related to the geometry of the dies and the sample cavity. The oscillation strain was varied between 0.01 and 40% during the test. The temperature could also be programmed to change at a rate of up to 1 °C, either upward or downward, within the range of 25 to 120 °C. For this investigation, a dynamic strain sweep was initially conducted to determine the linear viscoelastic (LVE) region by varying the RPA strain within the range of 0.01 to 40% at a constant frequency of 0.33 Hz and a temperature of 25 °C [36].

## 3. Results and Discussion

### 3.1. Preparation of SUFs

**Figure 2:**
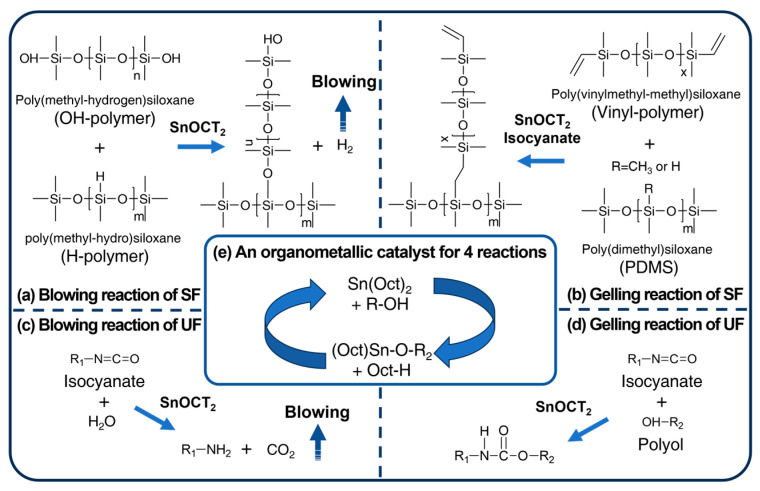
(**a**) Catalysts used for the foaming reaction, (**b**) blowing reaction of SF, (**c**) gelling reaction of SF, (**d**) blowing reaction of UF, and (**e**) gelling reaction of UF.

In the case of SF, the crosslinking and blowing reactions were conducted using Karstedt’s catalyst under an inert atmosphere, free from coordinating chlorides that can easily form at room temperature [37]. However, Karstedt’s catalyst is susceptible to deactivation by certain chemical compounds. In particular, nitrogen, phosphorus, and sulfur can interact strongly with the active sites of the catalyst, leading to poisoning. Consequently, even minute quantities of these catalyst poisons can significantly affect the adsorption of reactants on the catalyst. This phenomenon, known as catalyst poisoning, occurs when platinum in Karstedt’s catalyst is deactivated by nitrogen present in the isocyanate. In contrast, catalysts containing tin, such as Sn(OCT)_2_, are not affected by catalyst poisoning, allowing a hydrogen-generating self-foaming reaction to occur [38]. Figure 2a illustrates the hydrogen-generating self-foaming reaction between the hydroxyl-terminated and hydrogen-terminated polymers using Sn(OCT)_2_ instead of Karstedt’s catalyst or DBTL. This reaction successfully foams SF at room temperature with the catalyst present [39]. We also observed the crosslinking reaction between H-polymer and the vinyl-polymer facilitated by isocyanates. Isocyanates can generate free radicals at room temperature, and the reaction rate increases by 2.43 times at 98 °C [40]. Furthermore, isocyanates can be activated by organometallic catalysts, which enhances their reactivity [41,42]. The activated isocyanate produces free radicals that effectively react with the vinyl group at 100 °C. Figure 2b demonstrates this crosslinking reaction involving Sn(OCT)_2_ and isocyanates.

**Figure 3:**
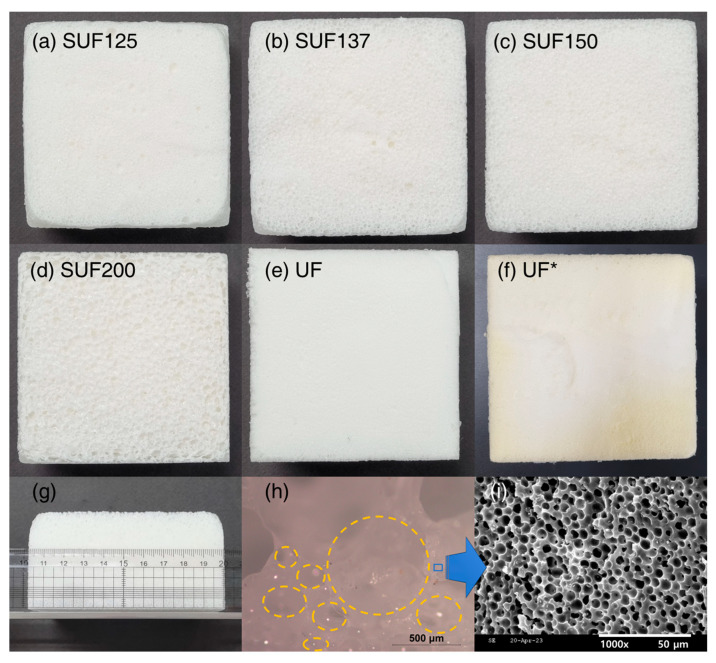
Digital photographs of SUFs with varying polyurethane contents: (**a**) SUF125, (**b**) SUF137, (**c**) SUF150, (**d**) SUF200, (**e**) UF, and (**f**) UF*. (**g**) illustrates the dimensions of the cone calorimetric test specimen (100 × 100 × 50 mm^3^), while (**h**) displays a digital microscopy image of the SUF137 cross-section and (**i**) an SEM micrograph of the SUF137 cross-section. (Yellow circles are pores created by CO_2_ foaming.).

The blowing reaction of UF (Figure 2c) occurs between isocyanate and H_2_O, forming carbamic acid, which then decomposes to produce CO_2_ and an amine group [43]. CO_2_ acts as the blowing agent, facilitating the foaming of the polymer. UF is a chemically blown foam that initially consists of a two-part mixture, with isocyanate in one part and polyol, water, surfactant, and catalyst in the other. The gelling reaction of UF (Figure 2d) results from a condensation reaction between isocyanate and polyol, leading to the formation of crosslinked polyurethane [43]. A key aspect of this study is that the four reactions of SF and UF occur simultaneously, resulting in SUF with Sn(OCT)_2_ participating in all these reactions (Figure 2e). For the formation of SUFs (Table 1), SUFs were prepared by mixing polyurethane at ratios of 125 to 200 parts by weight with 100 parts by weight of PDMS. SUFs can be produced by increasing the polyurethane content from 125 to 200, with Figure 3a–d indicating that cell size increases along with the amount of polyurethane. Figure 3e shows UF with a specific gravity of 0.06 g/cm^3^, prepared using FlexFoam-iT!™ III (Part A:Part B = 57.5:100). To compare the mechanical strength of SUF, Figure 3f depicts UF* with a specific gravity of 0.12 g/cm^3^, produced using FlexFoam-iT!™ IIIV (Part A:Part B = 100:100). The specimens measured 100 × 100 × 50 mm^3^ for the cone calorimeter test, as shown in Figure 3g. Figure 3h presents a digital microscopy image of a cross-section of the SUF, revealing pores of up to 1 mm formed due to CO_2_ foaming [44]. Additionally, Figure 3i displays an SEM micrograph of the SUF cross-section, showing that the pores formed by H_2_ were approximately 5 μm in size [45].

### 3.2. Characterization of SUFs

**Figure 4:**
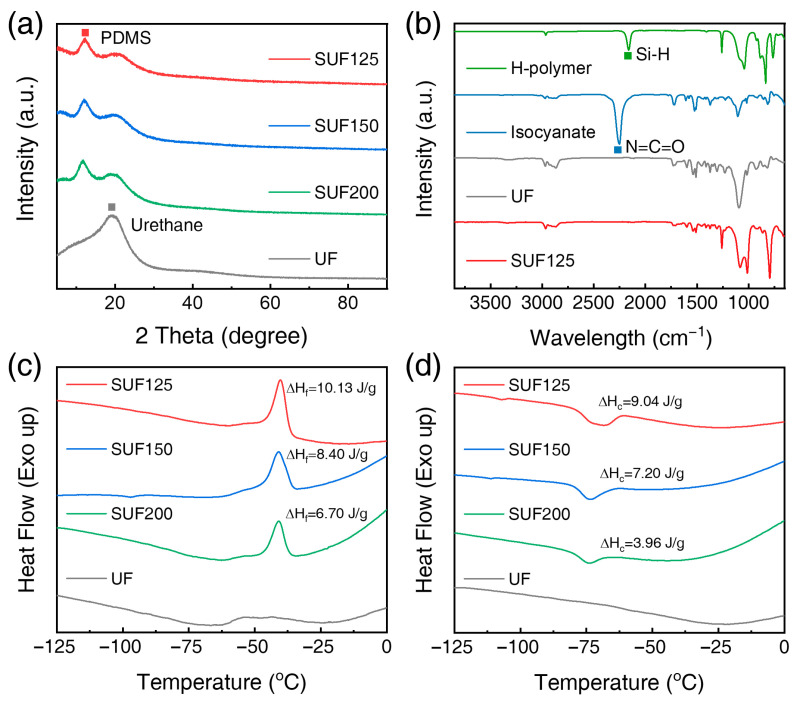
(**a**) XRD spectra of SUFs, (**b**) FT-IR spectra of SUF137, and DSC thermograms of the second (**c**) heating scan and (**d**) cooling scan for SUFs with varying polyurethane contents.

The XRD patterns of PDMS and UF, shown in Figure 4a, are consistent with previous studies [46,47]. The labeled peaks in Figure 4a indicate the PDMS phases within the SUFs; specifically, the SUFs exhibit a PDMS peak at 12.5° [46] and a polyurethane peak at 20° [47]. While UF only shows a polyurethane peak, the SUFs display both PDMS and polyurethane peaks. As the polyurethane content increased from 125 to 200 parts, the peak intensity correspondingly increased. Figure 4b presents the FT-IR spectrum of SUF137, which indicates a Si-H peak at 2162 cm^−1^ [48] and an isocyanate peak at 2252 cm^ࢤ1^ [49]. In the FT-IR spectrum of SUF125, Si-H at 2162 cm^ࢤ1^ [48] and isocyanate peak at 2252 cm^ࢤ1^ [49] were not detected, suggesting that the SUFs underwent complete reaction without deterioration of physical properties or combustion due to unreacted substances. DSC experiments were conducted to reveal thermal transitions and characterize the crystallization and melting behavior of the SUFs as a function of blend composition. Figure 4c,d present the DSC thermograms from the second heating scan of SUF composites; Figure 4c shows the heating scan while Figure 4d shows the cooling scan. The DSC thermograms were measured from –125 to 0 °C under a nitrogen atmosphere. Table 2 lists the crystalline melting point (*T_f_*), crystallization temperature (*T_c_*), enthalpy of fusion (Δ*H_f_*), enthalpy of crystallization (Δ*H_c_*), and *X_c_* of the SUFs. The DSC thermograms (values in Table 2) reveal that the introduction of polyurethane has minimal effect on *T_f_*; however, *T_c_* and *X_c_* of PDMS are influenced by the polyurethane content. The *X_c_* for SUF125 was 37.3%, which was higher than that of the other samples, but when the polyurethane content increased to 200 parts, *X_c_* decreased to 32.8%. The increase in Δ*H_f_* indicates a greater interaction among polymer molecules [50]. The Δ*H_f_* of SUF125 was 9.04 J/g, while the Δ*H_f_* decreased to 3.96 J/g when the polyurethane content increased to 200 parts. Based on the *X_c_* and Δ*H_f_* results, SUF125 exhibited the highest degree of *X_c_* for PDMS and the strongest interaction between polyurethane and PDMS.

**Figure 5:**
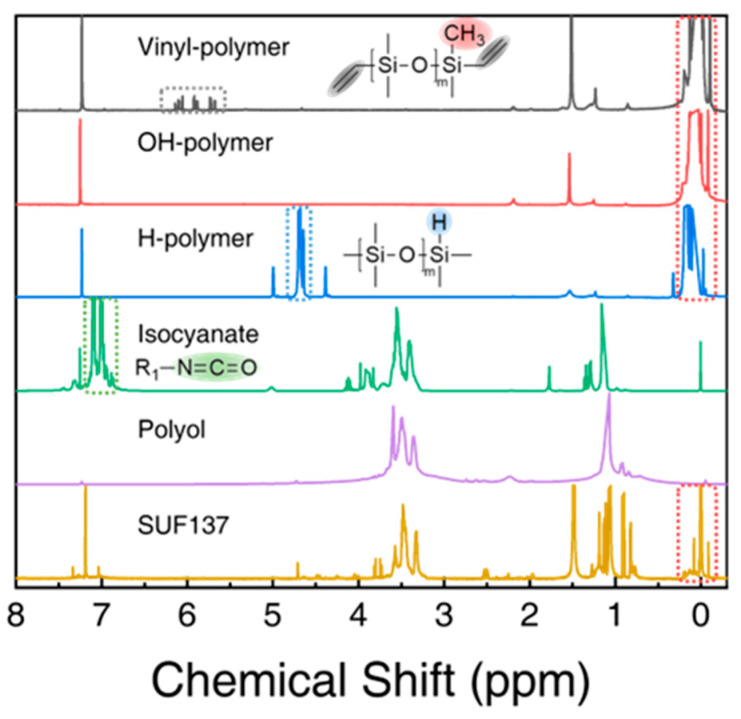
^1^H-NMR spectra of prepolymers and SUF137 with the inset showing the spectra of PDMS-vinyl, PDMS-H, and isocyanate.

To characterize the chemical structure of SUFs before and after the reaction, 1H–NMR was used to analyze vinyl-polymer and H-polymers. As shown in Figure 5, the peaks between 5.68 and 6.15 ppm correspond to vinyl structures [51], while the signals between 4.64 and 4.70 ppm are attributed to Si-H [52]. Peaks between 6.99 and 7.11 ppm indicate unreacted -NCO groups. The OH-polymer exhibited a hydroxyl peak at 2.19 ppm, while the hydroxyl peak in the polyol at 2.24 ppm was ascribed to hydrogen bonding. The polyol, which has a higher content of hydroxyl groups, is more susceptible to hydrogen bonding from the terminal hydroxyl groups of neighboring molecular chains, resulting in a shift to a lower field (2.24 ppm) where the peak broadened [53]. The curing of PDMS due to the reactions involving the vinyl groups caused the disappearance of the vinyl peaks in the ^1^H-NMR analysis of SUF137. This analysis confirmed that Sn(OCT)_2_ and isocyanate can function as curing catalysts for PDMS. Additionally, peaks for Si-H, -NCO, and -OH were not observed in SUF137. Consequently, 1H-NMR analysis demonstrated that SUFs were formed via four distinct reaction mechanisms.

### 3.3. Flammability and Characteristics of the Char Residues

**Figure 6:**
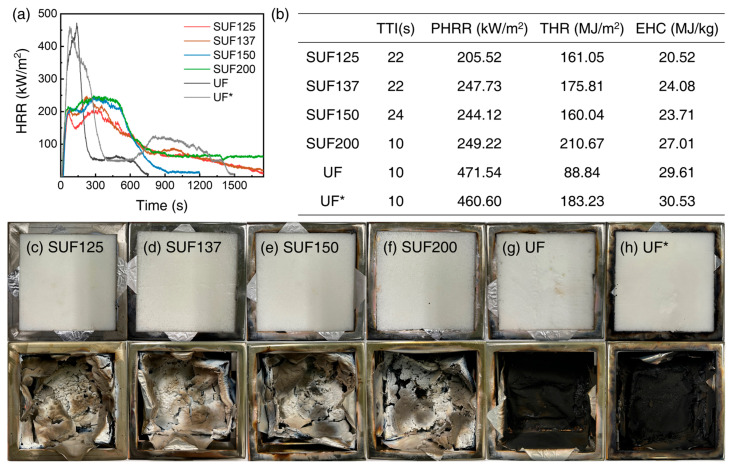
(**a**) HRR curves for the cone calorimeter test for SUFs with different urethane contents under the ISO 5660-1 standard with a heat flux of 25 kW/m^2^. (**b**) Key parameters obtained from cone calorimetry of SUF and digital photographs of SUFs with different urethane contents after the cone calorimetric test: (**c**) SUF125, (**d**) SUF137, (**e**) SUF150, (**f**) SUF200, (**g**) UF, and (**h**) UF*.

The flammability of the SUF in real fire conditions was further evaluated using a cone calorimeter. The HRR results are shown in Figure 6a,b. The UF with a specific gravity of 0.06 g/cm^3^ exhibited a PHRR of 471.54 kW/m^2^, while the UF* with a specific gravity of 0.12 g/cm^3^ displayed a PHRR of 460.60 kW/m^2^. The PHRR is influenced by the polyurethane content; specifically, when the content decreases from 200 to 125 parts, PHRR decreases from 249.22 to 205.22 kW/m^2^. The time to ignition (TTI) of the SUFs was more than twice as long as that of UF*, except for SUF200. The total heat release (THR) of SUF150 was lower than that of the other samples due to its specific density, while SUF125 exhibited a THR of 161.05 MJ/m^2^, attributed to its PDMS content. When the polyurethane content in SUFs ranged from 125 to 150 parts, TTI, PHRR, THR, and effective heat of combustion all improved compared to UF*.

Table 3 shows the total CO production (TCOP) and total CO_2_ production (TCO_2_P) of SUFs. The TCOP of SUFs is lower than that of UF*, and the TCOP of SUF137 is 1.02 g, which is lower than the others. The TCO_2_P of SUFs was reduced by 18.0 to 25.6% compared to that of UF*, and the TCO_2_P of SUF150 had the lowest value at 75.63 g. Because Si combines with O_2_ to form SiO_2_, the TCOP and TCO_2_P of the SUFs were lower than those of UF*. This reaction not only reduces toxic gases but also forms SiO_2_ ash layers with flame-retardant properties. Digital photographs before and after the cone calorimeter test are shown in Figure 6c–h, and the SiO_2_ ash layers are displayed in Figure 6c–f after the cone calorimeter test. In Figure 6g,h, UF and UF* left only black waste without ash layers.

### 3.4. Activation Energy and Mechanical Properties of SUFs

**Figure 7:**
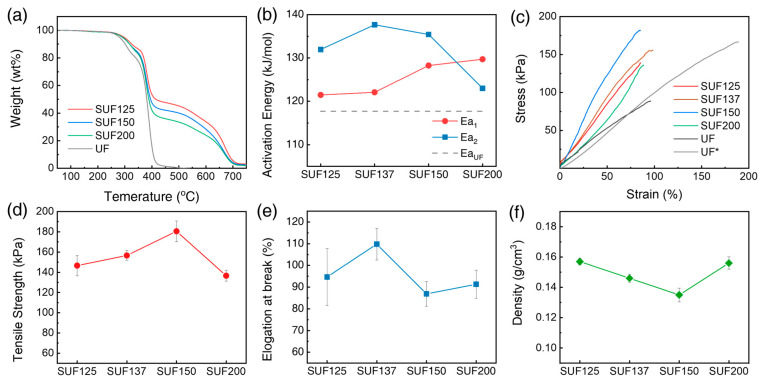
TGA curves were acquired for each polyurethane content at three heating rates (10, 15, and 20 °C/min). (**a**) TGA curves of SUF at a heating rate of 15 °C/min, (**b**) Ea of SUFs. TGA analysis was carried out under a nitrogen atmosphere (Ea_1_: The activation energy of SUF from 250 to 425 °C, Ea_2_: The activation energy of SUF from 425 to 725 °C, Ea_UF_: Activation energy of polyurethane), (**c**) Stress–strain curves of SUFs with different polyurethane contents. (**d**) Tensile strength, (**e**) elongation at break, and (**f**) density of SUFs at different urethane loadings with error bars. Error bars represent the standard deviation calculated from three samples, each with three replicates.

The curves and data regarding the thermal stability of the samples under a nitrogen atmosphere are depicted in Figure 7a,b. UF undergoes pyrolysis in the temperature range of 250–425 °C, while PDMS decomposes thermally between 425–725 °C. Consequently, SUFs exhibit two stages of weight loss during thermal degradation, corresponding to the pyrolysis of polyurethane and PDMS, respectively. In contrast, UF degrades earlier, displaying only one stage of weight loss. Figure 7a illustrates the TGA curve of SUFs at a heating rate of 15 °C/min, showing that thermal stability improves with increased PDMS content. In Figure 7b, the Ea of the SUFs was calculated using data from each heating rate (10, 15, and 20 °C/min) under nitrogen atmosphere via Equation (2). UF has a Ea_UF_ valued at 117.71 kJ/mol, whereas SUFs have two activation energies: Ea_1_ and Ea_2_. Ea_1_ pertains to polyurethane influenced by PDMS, and it is higher than Ea_UF_, increasing with urethane content. Ea_2_, measured after polyurethane pyrolysis, is affected by interactions with PDMS. The Ea_2_ value of SUF137 is 137.65 kJ/mol, higher than that of other samples. The interaction of polymer chains in SUFs is reflected in both Ea1 and Ea_2_, with SUF150 exhibiting the strongest polymer chain interaction, followed by SUF137, SUF125, and SUF200.

In previous studies, conventional UF reduced flame hazards by incorporating flame-retardant fillers. However, flame-retardant UF composites typically exhibit weak interactions between the polymer chains and fillers, leading to decreased tensile strength and compressive strain [54]. In contrast, SUFs, which blend PDMS instead of flame-retardant fillers, are expected to offer enhanced flame-retardant properties and superior mechanical strength. The mechanical properties of SUFs were evaluated using tensile strength tests and 500-cycle compression–release tests at 75% compression strain. Figure 7c presents the stress–strain curves of SUFs with varying polyurethane contents from 125 to 200 parts. The tensile strength and elongation at break values are detailed in Table 4. Notably, the tensile strength of SUF150 increased by 8.4%, despite the addition of flame-retardant materials (Figure 7d). Figure 7e illustrates the elongation at break, showing SUF composites achieving 94.7–109.8%, approximately half of UF* and similar to UF. As depicted in Figure 7f, the specific gravity of SUFs decreased from SUF125 to SUF150 with increasing polyurethane content due to improved blowing reactions; however, the specific gravity of SUF200 increased due to rapid gelling reactions.

**Figure 8:**
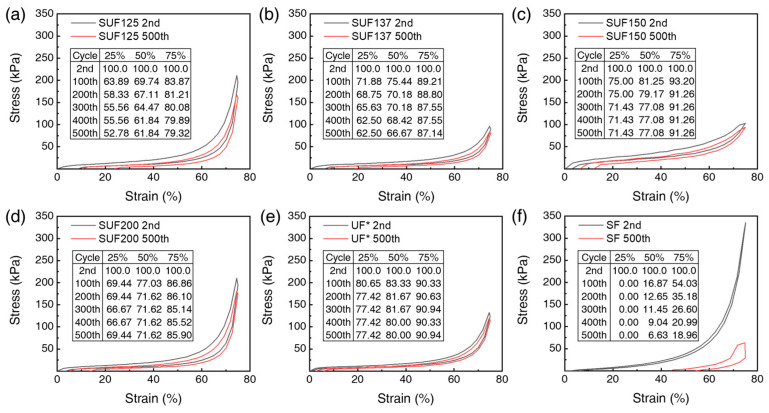
Compression test results of SUF with different urethane contents: (**a**) SF, (**b**) SUF125, (**c**) SUF137, (**d**) SUF150, (**e**) SUF200, and (**f**) UF*. (Extrapolated 2nd and 500th cycle test results.) The inset shows the results of 2nd, 100th, 200th, 300th, 400th, and 500th repetitions at 25, 50, and 75% strain.

As shown in Figure 8a–d, cyclic compression tests were conducted on the SUFs to assess durability, summarizing the results from the 2nd to the 500th cycle. The compressive stress of the SF showed more than a 50% stress loss at 75% strain and exhibited no stress at 25% strain after 100 compression cycles. In contrast, the unloading curve of SUF150 nearly returned to its original state even after 500 cycles at 75% compressive strain, demonstrating excellent elasticity and superior shape recovery without plastic deformation after pressure release. SUF150 showed only an 8.74% compressive stress loss after 500 cycles, confirming its superior elasticity and flexibility compared to UF*, which had a 9.06% loss (Figure 8e). For comparison with SUFs, the results of repeated compression tests of SF are shown in Figure 8f. These outstanding mechanical properties suggest that SUFs are suitable for applications in automobile seats and chairs.

### 3.5. Rheological Properties of SUF

**Figure 9:**
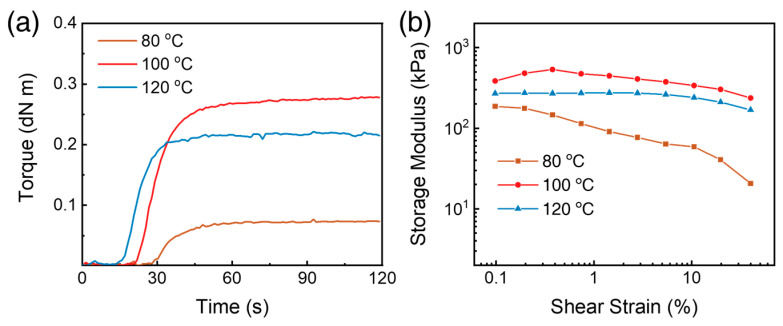
RPA results of SUF137 with different curing temperatures: (**a**) Torque curves of SUF 137 during curing and (**b**) variation of storage modulus under dynamic shear strain sweep.

To investigate the effect of curing temperature on SUFs using RPA, the curing curves of SUF137 were plotted at various temperatures. Figure 9a illustrates the torque changes of SUF137 during curing at different temperatures, and Table 5 lists the minimum torque (ML), maximum torque (MH), and t_90_ (90% curing time) for the SUFs. As shown in Figure 9a, SUF137 exhibited insufficient crosslinking at 80 °C but demonstrated the highest torque at 100 °C. Although t_90_ decreased as the curing temperature increased, the curing temperature for SUF was set at 100 °C to optimize torque. The dependence of the storage modulus (*G*′) on strain amplitude for SUFs with different curing temperatures is illustrated in Figure 9b. When the curing temperature exceeded 100 °C, SUF137 displayed linear reductions in *G*′ with increasing strain amplitude.

**Figure 10:**
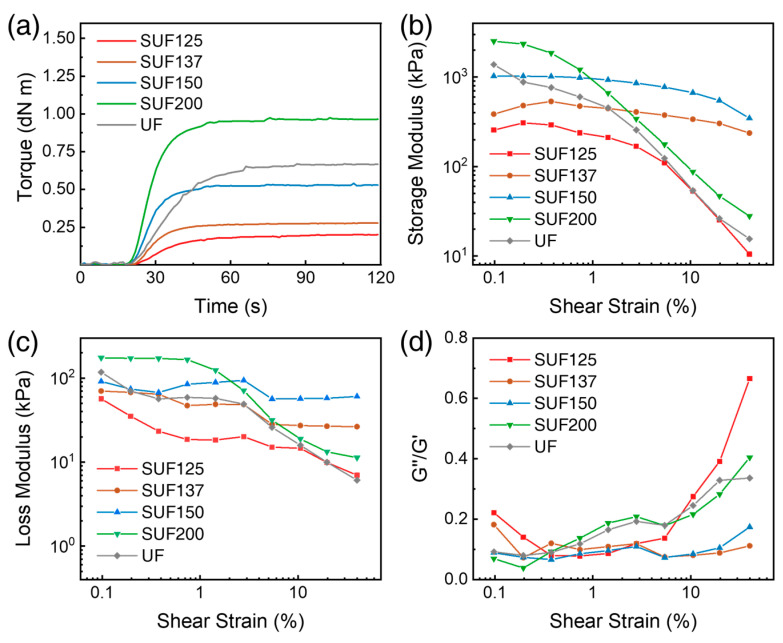
RPA results of SUF with different polyurethane contents: (**a**) Torque curves of SUF composites during curing at 100 °C, (**b**) variation of storage modulus under dynamic shear strain sweep, (**c**) variation of loss modulus under dynamic shear strain sweep, and (**d**) variation of G″/*G*′ under dynamic shear strain sweep.

To analyze the effect of polyurethane content on SUFs using RPA, the torque curves of SUFs with different polyurethane contents are displayed in Figure 10a. As the polyurethane content increased, the dynamic curing torque and speed of the SUF increased. Figure 10b shows the *G*′ values of the SUFs with different polyurethane contents. SUF137 and SUF150 showed linear reductions in *G*′ in strain amplitude, and *G*′ was constant even at 40% strain because of the strong interaction between vinyl-polymers. SUF125 has poor *G*′ and weak interaction between vinyl-polymers due to insufficient radicals by low isocyanate content. SUF200, which has the lowest PDMS content, displays a nonlinear reduction in *G*′ and has a poor *G*′ at 40% strain, similar to UF.

To analyze the effect of polyurethane content on SUFs using RPA, the torque curves of SUFs with varying polyurethane contents are presented in Figure 10a. As the polyurethane content increases, both the dynamic curing torque and the speed of the SUFs increase. Figure 10b illustrates the *G*′ values of the SUFs with different polyurethane contents. SUF137 and SUF150 exhibit linear reductions in *G*′ with increasing strain amplitude, maintaining constant *G*′ even at 40% strain due to strong interactions among the vinyl-polymers. In contrast, SUF125 shows lower *G*′ values and weaker interactions among the vinyl-polymers, attributed to insufficient radicals resulting from low isocyanate content. SUF200, which contains the lowest PDMS content, displays a nonlinear reduction in *G*′ and exhibits low *G*′ at 40% strain, similar to UF.

(5) $$\sigma =\frac{E^{\prime }}{3RT}$$

(6) $$E=2\left(1+v\right)G$$

The storage modulus at the rubbery plateau (*T* > *Tg*) is proportional to the crosslink density [54]. Equation (5), which describes the modulus of elasticity, was proposed by Lakshimi et al. [54,55,56,57]. In Equation (5), σ represents the crosslink density of the SUFs, *R* is the gas constant, *T* is the absolute temperature, and *E*′ is the elastic modulus of the SUFs in the rubbery plateau region [54,58]. According to Equation (6), the Poisson ratio (*v*) ranges from 0.49 to 0.499 for elastomers, implying that the elastic modulus is three times the storage modulus. Thus, *G*′ and crosslink density are directly proportional to each other. The effective crosslink density accounts for both chemical and physical crosslinks [59], and chain entanglements can enhance effective crosslinking [60]. Due to the high crosslinking density, SUF137 and SUF150 exhibit high *G*′ values, as well as excellent mechanical properties and flame-retardant performance. As shown in Figure 10c, SUF137 and SUF150 exhibited linear reductions in the *G*″ with increasing strain amplitude. To assess durability through repeated compression tests, the *G*″/*G*′ of the SUFs are illustrated in Figure 10d. SUF137 and SUF150 demonstrate excellent durability compared to UF due to their low *G*″/*G*′ values at 40% strain. Conversely, the *G*″/*G*′ value for SUF125 at 40% strain is 0.67, which is higher than that of the other samples due to its low crosslink density. The *G*″/*G*′ value for SUF200, which has the lowest PDMS content, is 0.40, lower than UF’s value of 0.34.

## 4. Conclusions

In summary, we developed a practical method for preparing SUFs, in which the vinyl-polymer is cured using Sn(OCT)_2_. The FT-IR spectra of SUF137 demonstrated successful crosslinking reactions, as evidenced by the absence of the Si-H peak at 2162 cm^−1^ and the -NCO peak at 2252 cm^−1^. Additionally, the disappearance of the vinyl peak in the ^1^H-NMR spectrum between 5.68 and 6.15 ppm further confirmed these crosslinking reactions. The differences in the properties of the SUFs were examined using XRD and DSC, while the activation energy was calculated from TGA data to establish a correlation between the Ea and mechanical properties of the SUFs. Results from repeated compression tests and tensile strength tests indicated that SUFs exhibit substantially improved durability compared to SF, with the durability of SUF150 comparable to that of UF*. In the cone calorimeter test, the PHRR of SUF125 decreased by 56.42% compared to UF. Furthermore, TCOP and TCO_2_P production were reduced as oxygen combined with silicon during combustion to form SiO_2_. Rheological analysis using RPA revealed the optimal curing temperature of the SUFs as well as their elastic properties, which contribute to the high durability and excellent mechanical properties of SUF137 and SUF150. This novel and efficient manufacturing method for SUFs offers a new approach to minimizing the generation of flame-hazardous and toxic gases from widely used materials.

## Figures and Tables

**Table 1:** Formulations for the preparation of SUFs.

Samples	PDMS			Catalyst	Polyurethane (FlexFoam-iT!™ III)	
	Vinyl Polymer	OH Polymer	H Polymer	Sn(OCT)_2_	Part A	Part B
SUF125	30	65	5	0.1	46	79
SUF137	30	65	5	0.1	50	87
SUF150	30	65	5	0.1	55	95
SUF200	30	65	5	0.1	73	127

**Table 2:** Key parameters obtained from DSC of SUFs.

	*T_f_* (°C)	*T_c_* (°C)	${∆}_{f}H$ (J/g)	${∆}_{c}H$ (J/g)	*X_c_* (%)
SUF125	−40.24	−68.52	10.13	9.04	37.3
SUF137	−40.61	−70.75	9.28	8.75	35.9
SUF150	−40.94	−73.75	8.40	7.20	34.3
SUF200	−40.94	−73.78	6.70	3.96	32.8

**Table 3:** Key parameters obtained from cone calorimetry of SUFs.

	TCOP (g)	TCO2P (g)
SUF125	1.08	80.45
SUF137	1.02	82.22
SUF150	1.10	75.63
SUF200	1.69	88.41
UF*	2.26	100.24

**Table 4:** Mechanical properties and density of SUFs.

	Tensile Strength (kPa)	Elongation at Break (%)	Density (g/cm^3^)
SUF125	146.6 (±10.1)	94.7 (±13.1)	0.157 (±0.0022)
SUF137	156.6 (±4.9)	109.8 (±7.3)	0.146 (±0.0026)
SUF150	180.5 (±10.3)	86.9 (±5.7)	0.135 (±0.0045)
SUF200	136.6 (±5.3)	91.3 (±6.5)	0.156 (±0.0041)
UF	86.4 (±5.9)	101.1 (±3.4)	0.060 (±0.0015)
UF*	166.5 (±8.7)	189.2 (±20.3)	0.120 (±0.0032)

**Table 5:** Average curing properties and moduli of SUFs.

	ML (dN m)	MH (dN m)	∆M (dN m)	t_90_ (s)
SUF125-100 °C	0.001	0.204	0.203	63.8
SUF137-100 °C	0.001	0.279	0.278	44.6
SUF150-100 °C	0.001	0.544	0.543	43.4
SUF200-100 °C	0.001	0.974	0.975	42.2
UF-100 °C	0.002	0.669	0.667	59.0
SUF137-80 °C	0.001	0.077	0.076	53.0
SUF137-120 °C	0.001	0.222	0.221	33.8

## Data Availability

Data is contained within the article.
